# In Vitro Protein Digestibility of Selected Seaweeds

**DOI:** 10.3390/foods11030289

**Published:** 2022-01-21

**Authors:** Goldy De Bhowmick, Maria Hayes

**Affiliations:** Food BioSciences Department, Teagasc Food Research Centre, D15 KN3K Dublin, Ireland; Goldy.DeBhowmick@teagasc.ie

**Keywords:** in vitro protein digestibility, k-PDCAAS, seaweeds, essential amino acids, limiting amino acids

## Abstract

Seaweed biomass is considered a valuable and potential, alternative protein source but it is currently under-exploited. Seaweed or Macroalgae do not require arable land and freshwater for their cultivation, they are fast growing and contain several health ingredients and beneficial macronutrients. In this study, we determined the in vitro k-Protein Digestibility-Corrected Amino Acid Score (k-PDCAAS) values of six different, Irish seaweeds using the rapid k-PDCAAS method. Based on the amino acid profile and protein content of each seaweed, the in vitro protein digestibility and k-PDCAAS scores were calculated. In addition, the limiting amino acid(s) for each of the six seaweeds was/were determined. Results suggest that although the in vitro digestibility was quite similar for all analyzed seaweeds, their k-PDCAAS scores varied significantly. The red seaweed *Palmaria palmata* had a k-PDCAAS score of 0.69 ± 0.014, while *Fucus serratus* had a value of 0.63 ± 0.084 and *Alaria esculenta* a value of 0.59 ± 0.021. The seaweeds were found to be rich in essential amino acids and taurine. Overall, the amino acid composition of the seaweeds studied suggests that they are suitable alternative protein sources for use in human nutrition providing both essential and non-essential amino acids to the consumer.

## 1. Introduction

An increased demand for food production in order to feed the growing population is a key driver in the search for innovative food ingredients [1,2]. Additionally, arable land and freshwater cannot facilitate current demand for proteins [3,4]. At present, marine natural resources are under-exploited [3,4,5]. Seaweeds, also known as macroalgae, do not require terrestrial land and freshwater for their cultivation, they are fast growing and contain several health ingredients and beneficial macronutrients [3,6]. There is a long history of seaweed use in Asian diets but the popularity of seaweeds or sea vegetable consumption in Western society is also growing [2,5,7]. The global seaweed market size was $4097.93 million in 2017 and is projected to reach $9075.65 million by 2024, creating many opportunities for innovative food product development [2,8]. The European Commission support blue growth initiatives encouraging the ‘Farm to Fork Strategy’ and are actively promoting consumption of more algae [2,8,9]. Many studies related to the incorporation of seaweed ingredients into breads, cereals and other food products significantly increase the nutritional quotient of the developed products [1,2,10].

Several studies have shown that seaweeds contain significant levels of protein and essential amino acids (EEAs) [9,11,12]. Usually, the protein concentration in seaweeds varies from 5 to 47% (red seaweeds: 10 to 30%; brown seaweeds: 5 to 15%; and green seaweeds: 3 to 47%) on a dry weight basis but their composition depends on the species (both within and between species) and cultivation environment [11,12,13]. Abiotic conditions in the marine environment are highly variable, which is related to seasonal and geographical variation that primarily affects the physiological performance (growth and reproduction) and biochemical composition of seaweeds [3]. Such variation in environmental conditions results in variation in protein content and composition [3]. Additionally, the EEA composition of selected seaweed proteins represents half of the total amino acid (TAA) requirements recommended for humans by the Food and Agriculture Organization of the United Nations (FAO) [11,12]. Moreover, amino acids, especially glycine, alanine, arginine, proline, glutamic acid and aspartic acid, are abundantly found in red, brown and green seaweeds [11,12]. Bleakley and Hayes, (2017) previously reported that algal proteins are often on par with other protein sources including soy and egg protein in terms of amino acid content (e.g., 22–44% of total amino acids in *Fucus* sp). However, due to the lack of widespread consumption of seaweeds, a deficit in understanding the bioaccessibility and bioavailability of algal proteins exists [14]. Furthermore, the PDCAAS values for several seaweed species are currently not reported in the literature. It is important to know this value as it is an indicator of how bioavailable amino acids are and it is indicative of the content of anti-nutritional factors such as saponins, tannins and lectins in seaweed biomass [15,16,17].

The type and nutritional quality of proteins present in foods impacts on digestion, absorption and the potential health impacts of proteins and amino acids. According to the FAO, protein quality is related to amino acid composition and bioavailability, which is directly proportional to the digestibility of the ingested proteins [14,18]. Protein quality is usually determined in costly animal studies using pig and rat models [5,19]. The in vivo Protein Digestibility-Corrected Amino acid score (PDCAAS) was considered the “gold standard” for protein quality determination but was recently replaced by the Digestible Indispensible Amino Acid Score (DIAAS) method [20]. However, methods are costly and in vitro methods are useful to guide selection of proteins for use in these in vivo models and to reduce costs in finding suitable, alternative proteins. It is considered that the nutritive value of protein is equal to the biological value (BV) of the protein multiplied by the true digestibility (D) of the same protein that is referred to net protein utilization (NPU) [5]. If proteins are viewed as primary sources of indispensable amino acids then the quality of a given protein rich food as a source of DIAAS is also very important [20].

In spite of FAO recommendations, the PDCAAS values of seaweed proteins are either scanty or largely unknown [12]. The aim of this paper is to determine the in vitro PDCAAS values of six different Irish seaweeds using the rapid k-PDCAAS method [21]. The k-PDCAAS assay kit is used for in vitro measurement of animal safe accurate protein quality score method developed by Medallion labs. This digestibility score in conjunction with essential amino acid profile plus protein content is used to calculate the PDCAAS value. Based on the amino acid profile and protein content of each seaweed, the in vitro protein digestibility and k-PDCAAS scores were calculated for the individual seaweeds. In addition, the limiting amino acids for each of the six seaweeds was determined. Results suggest that although the in vitro digestibility was quite similar for all analyzed seaweeds, their k-PDCAAS score varied significantly. The red seaweed *Palmaria palmata* had the highest k-PDCAAS score, followed by *Fucus serratus* and *Alaria esculenta*.

## 2. Materials and Methods

### 2.1. Six Seaweeds

The following seaweeds were supplied by the Irish companies SeaLac Ltd. (Sligo, Ireland) and Arramara teoranta (Connemara, Galway, Ireland)—*Alaria esculenta*; *Fucus serratus*; *Fucus vesiculosus*; *Ulva lactuca*; *Palmaria palmata* and *Asparagopsis taxiformis*.

### 2.2. Analytical Procedures

#### 2.2.1. Protein Content

Protein concentration was determined using a LECO FP628 protein analyzer (LECO Corp., Benton Harbor, MI, USA) according to the AOAC nitrogen determination by combustion method (Dumas Method), Official method 992.15 (1990). Biancarosa and colleagues reported the nitrogen-to-protein conversion factor for different seaweed proteins. Thus, a nitrogen-to-protein conversion factor of 5.13 ± 0.1 for brown, 3.99 ± 0.39 for red, and 4.24 ± 0.46 for green algae were used to determine the crude protein content and the proteins were expressed as a percentage value based on three independent analysis [22]. The percentage nitrogen as determined by LECO FP628 was multiplied by the nitrogen conversion factor to determine the percentage protein of each sample.

#### 2.2.2. Amino Acid Profile Analysis

Briefly, the total amino acid composition of the seaweed samples was determined by using hydrolysis with 6 M HCL at 110 °C for 23 h. Approximately, 8–10 mg of dried samples was used for hydrolysis and dried in a vacuum centrifuge. The samples were then deproteinized by mixing equal volumes of 24% (*w*/*v*) tri-chloroacetic acid and sample. Samples were allowed to stand at room temperature for 10 min before centrifugation at 14,400 × *g* for 10 min. The supernatants were recovered and diluted with 0.2 M sodium citrate buffer, pH 2.2, to give approximately 250 nM of each amino acid residue. Samples were then diluted 1:2 with the internal standard nor-leucine to give a final concentration of 125 nM/mL. Amino acids were quantified using a Jeol JLC-500/V amino acid analyzer (Jeol Ltd., Garden city, UK) fitted with a Jeol Na+ high performance cation exchange column. Xell AG, Germany, carried out the amino acid analysis.

#### 2.2.3. Protein Digestibility-Corrected Amino Acid Score (PDCAAS) Method (Megazyme, Wicklow, Ireland)

The protein quality of the developed feeds was determined by assessing the amino acid composition of each in addition to using the in vitro Protein Digestibility-corrected amino acid score (PDCAAS) enzyme digestion method, a Megazyme assay kit according to the manufacturer’s instructions (Megazyme, Wicklow, Ireland). Briefly, protein samples were digested sequentially as previously described [21] using pepsin, trypsin, and chymotrypsin at neutral pH and undigested proteins were removed by precipitation with trichloroacetic acid (TCA). 2% Ninhydrin solution was used to quantify the α-amino acid concentration present in the sample with respect to L-glycine standards and the absorbance was recorded at 570 nm. Subsequently, the three feed samples were milled using a ball mill (Mixer mill MM 400; Retsch; Ireland) and 500 mg of each milled samples was used for the assay. 19 mL of HCl (0.06 N) were added to each of the samples and the sample was incubated for 30 min at 37 °C in a shaking incubator (Maxq 8000; model no: 444; serial no: 185550-31; Thermo Fisher Scientific; Dublin, Ireland) set at 150 rpm. 1 mL of the provided pepsin solution was added to each of the samples and vortexed and incubated for an hour at 37 °C in a shaker incubator set at 150 rpm. After pepsin incubation, samples were removed and the pH was adjusted to 7.4 by adding 2 mL of 1.0 M Tris buffer, pH 7.4. Samples were thoroughly mixed using a vortex and 200 μL of trypsin/chymotrypsin was added to each sample. Samples were vortexed and incubated for 4 h at 37 °C in the incubator set at 150 rpm. At the end of the trypsin/chymotrypsin digestion, the samples were placed in a boiling water bath for 10 min. Following incubation the samples were removed from the water bath and vortexed. Subsequently, the samples were completely cooled to room temperature for 20 min and 1 mL of 40% TCA solution was added to the mixture. Samples were incubated at 4 °C overnight. Following overnight incubation samples were centrifuged for 10 min at 15,000 rpm at room temperature. 10 to 20 fold dilutions in acetate buffer (50 mM, pH 5.5) of each sample were prepared prior to colorimetric assay.

### 2.3. Calculations

PDCAAS values were calculated using the Megazyme Mega-CalcTM programme (K-PDCAAS Mega-Calc) available from the Megazyme website (https://support.megazyme.com/support/solutions/articles/8000062829-protein-digestibility-k-pdcaas-mega-calc, accessed on 10 October 2021) and from the equations as described below.

(a) Primary amine concentration:

The L-glycine standard curve was generated using the absorbance values (*y*-axis) recorded at 570 nm by plotting against L-glycine concentration (x-axis) from 0 to 1 mM. Subsequently, the primary amine concentration (CI) of unknown samples were calculated using the following equation:

primary amine concentration (mM), Y = A × CI + B(1)

where

CI = unknown concentration of the primary amines (mM),
Y = absorbance,
B = y-intercept and
A = slope of the line.

(b) Primary amine concentration corrected for dilution and weight

Primary amine concentration in the original sample solution (C2) was calculated by multiplying the values obtained from Equation (1) by the dilution factor and also adjusting for any deviation from nominal sample size as follows:

C2 = C1 × D × 1.25 × (0.5)/W(2)

where

CI = concentration of primary amines in the diluted samples,
D = dilution factor of the samples prior to amine determination,
1.25 = dilution with TCA (all samples equal),
W = sample weight (g), and
0.5 = nominal size (g).

(c) Primary amine concentration corrected for amino acids present

Various amino acid constants were used to calculate the corrected primary amine concentration (CN) for the amino acids present as follows:

CN = C2 + [(Prol × 2 × 10)/(Lys × 0.5 × 10)] + (Hist × 0.2 × 10) + (Arg × 0.2 × 10)(3)

where

C2 = corrected primary amine concentration in the original sample solution (mM),
Prol, Lys, Hist, and Arg = concentration of L-proline, L-lysine, L-histidine, and L-arginine, respectively, in the original sample, and
2, 0.5, 0.2 and 0.2 = constants for various amino acids.

(d) In vitro digestibility

Firstly, using the literature values for the rat model, a data fit comparison was created for corrected primary amine concentration (CN) by a linear regression equation. Using the equation from the data fit, in vitro digestibility of the samples were calculated as follows:

in vitro digestibility = (M × X + B)/100(4)

where

X = corrected primary amine concentration for each sample,
M = slope of the line,
B = y-intercept, and
100 = conversion from percentage to grams.

(e) Determination of amino acid ratio and limiting amino acid

To determine the amount of each amino acid present in each sample, separate, total crude protein results were generated on a g/100 g of protein basis following a reference recommended as follows:

AA(g/100 g) = (AA g/100 g sample)/(crude protein%)(5)

Ratio = (mg/protein in sample)/(mg/g protein in reference sample)(6)

(f) Determination of in vitro PDCAAS score, and
(g) The in vitro PDCAAS score was calculated by multiplying the in vitro digestibility from Equation (4) by the limiting amino acid ratio (lowest value) from Equation (6).

PDCAAS = Digestibility ∗ Ratio
where
PDCAAS = in vitro PDCAAS score, Digestibility = in vitro digestibility from Equation (4), and
Ratio = ratio of limiting essential amino acid from Equation (6).

### 2.4. Statistical Analysis

All the experiments were performed in triplicates, and the results of this study were expressed as the mean ± standard deviation (SD) of the two replicates. Additionally, one-way ANOVA and *p* values were determined for all the six seaweed samples. Furthermore, a comparison test between the species for k-PDCAAS was also performed using the Tukey–Kramer procedure. Excel ANOVA package was used for the statistical analysis.

## 3. Results and Discussions

### 3.1. Seaweeds and k-PDCAAS Assessment

Three brown (*A. esculenta*, *F. serratus*, and *F. vesiculosus*), one green (*U. lactuca*), and two red (*P. palmata* and *A. taxiformis*) seaweeds were used in this study. The nutritional value and health benefit of these seaweeds, particularly *A. esculenta*, *F. serratus*, *F. vesiculosus*, *U. lactuca* and *P. palmata*, in the human diet are well documented [23,24]. These seaweeds are rich in minerals, vitamins, natural antioxidants and are often highlighted as functional foods [23,24]. Previous studies outlined the protein content and quality of these seaweeds [23,24], therefore it would be beneficial to have the protein in vitro digestibility of these seaweeds. In this study, the k-PDCAAS in vitro method was used to evaluate the protein quality of six different seaweeds based on human amino acid requirements as outlined by the FAO and human digestibility of the same [25,26]. The in vitro digestibility, amino acid score, and k-PDCAAS and crude protein percentage values of six seaweed samples are illustrated in Table 1. Amino acid profiles for each of the six seaweed samples are presented in Table 2. The ratios of the amino acid profiles were calculated based on the reference protein following Equation (6) or by using the Mega-CalcTM package (k-PDCAAS Mega-Calc) of the k-PDCAAS calculator [21]. The red seaweed *P. palmata* had a k-PDCAAS score of 0.69 ± 0.014 followed by the brown seaweeds specifically *F. serratus* with a k-PDCAAS score of 0.63 ± 0.084 and *A. esculenta* which had a score of 0.59 ± 0.021. The amino acid score in combination with protein digestibility is a method to determine the completeness of the proteins that are being ingested and are not fecal [20]. The amino acid score was also found to be the highest for *P. palmata* ~0.883 ± 0.019, followed by *F. serratus* and *A. esculenta* ~0.825 ± 0.108 and 0.751 ± 0.022. Although the amino acid score and k-PDCAAS were significantly different to each other for each, individual seaweed, the in vitro digestibility of the seaweeds were similar to each other. Similarity in in vitro digestibility was due to the corrected primary amine concentration, which was found to be similar for all the seaweeds. The brown seaweed *F. vesiculosus* had the highest in vitro digestibility value ~0.82 ± 0.001 but had a calculated k-PDCAAS value of 0.08 ± 0.013. This k-PDCAAS value is due to the difference in the amino acid concentrations determined for this seaweed with respect to the reference protein ratio considered in this method (i.e., casein). Casein, was used as a standard protein and the in vitro digestibility obtained for casein was observed as 1.00 as observed with previous studies [27,28]. k-PDCAAS values range from 1 to 0, wherein 1 represents protein sources with a high biological value such as milk or eggs that provide the indispensable amino acids recommended by FAO [28,29]. The greater the k-PDCAAS the better the biological value of the food protein source. A k-PDCAAS score close to 1.00 means that the protein source can supply all the amino acids required by the human body for optimum nutrition [12,21].

Protein quality is a measure of its digestibility and bioavailability and is affected by many factors such as the amino acid composition of the food, food preparation methods and treatment processes applied to the food and the granularity of the food product [30]. Here, food preparation including treatment process and granularity can be discarded as dried seaweed biomass was tested; hence, amino acid composition was given significant importance. In this study, the k-PDCAAS values observed for *P. palmata*, *F. serratus* and *A. esculenta* suggest that the bioavailability of protein in these seaweeds is greater than protein found in *A. taxiformis*, *U. lactuca* and *F. vesiculosus*. Additionally, it was observed that even though the crude protein value observed for *F. vesiculosus* was greater than that observed for *F. serratus*, *F. vesiculosus* had a lower k-PDCAAS value. Seaweeds containing higher protein percentage values are not necessarily more bioavailable. Plant legumes, wheat, vegetables and fruits have k-PDCAAS values ranging from 0.4 to 0.7, whereas soy, milk, chicken, fish and beef have k-PDCAAS values of between 0.8 and 1 [30]. The k-PDCAAS values for *P. palmata*, *F. serratus*, and *A. esculenta* are equivalent to those recorded previously for wheat (PDCAAS: 0.42), barley (PDCAAS: 0.44–0.53), fruits and vegetables (PDCAAS: 0.73–0.64), and chickpea (PDCAAS: 0.62–0.65) proteins, demonstrating that they can be considered suitable protein sources comparable to plant proteins [31,32,33].

The green seaweed *Ulva* sp. has a reported protein content of 7–24% based on its’ dry weight [34]. However, from our study it can be observed that the protein content of *U. lactuca* recorded at 5.37 ± 0.74% is lower than that recorded previously and this seaweed had a k-PDCAAS value of 0.15 ± 0.014, which is less than the values observed from red and brown seaweeds in this study. This value may be due to the geographic and environmental conditions at the time of harvest. Typically, a nitrogen-to-protein conversion factor of 6.25 is used for protein% calculation; however, this approach can be inaccurate when applied to seaweeds resulting in overestimation of their protein content [22]. The nitrogen conversion factors mentioned by Biancarosa et al. (2017) were applied in this study [22]. Furthermore, the total amino acid contents of each seaweed were determined. Additionally, Gilan et al. (2005), reported that the presence of certain compounds such as trypsin inhibitors and hemagglutinins in legumes, tannins in legumes and cereals, phytates in cereals and oilseeds, and gossypol in cottonseed protein products have been shown to adversely affect the protein digestibility and amino acid availability [35]. Moreover, it was observed previously that the presence of high levels of tannins in cereals such assorghum and grain resulted in significant reduction (up to 23%) of protein and amino acid digestibility in rats, poultry, and pigs. Therefore, high levels of certain bioactive compounds in the tested seaweeds may have resulted in lower k-PDCAAS value. For example, the presence of high levels of *Ulvans* in *U. lactuca*, bromoform in *A. taxiformis*, and tannins in *F. vesiculosus* may have negatively affected the k-PDCAAS values for these seaweeds. Recently, Barbosa et al. (2020) evaluated the phlorotannin content of different Fucus species and found that F. vesiculosus had a greater phlorotannin content than F. serratus, hence such differences in phlorotannin content maybe one of the reason for such fluctuation in the observed k-PDCAAS values [36]. Cian et al. (2014), reported the k-PDCAAS value of 0.327 (32.7%) for the red seaweed *Porphyra columbina* [29] previously—a value that is greater than that reported for A. taxiformis (k-PDCAAS ~ 0.31 ± 0.01) but lower than the k-PDCAAS for P. palmata (0.69 ± 0.014) observed in our study. In another study, Cian et al., (2014b) reported protein digestibility of *P. columbina* at 0.743 ± 0.03 which was similar to our findings where we observed the in vitro digestibility of the *P. palmata* and *A. taxiformis* to be 0.78 ± 0.002 and 0.79 ± 0.001, respectively [37]. As most of the studies on seaweed proteins are reported as either protein percentage values or amino acid compositions there were no reports available on the k-PDCAAS for the six seaweeds assessed here, therefore, a direct comparison on protein bioavailability cannot be performed. However, we compare the values obtained in this study with other plant based protein sources as shown in Table 3.

The k-PDAAS analysis helps us to understand that protein quality should also be accounted for in terms of food use and sustainability. One-way ANOVA was also performed between the seaweed samples for the amino acid score and k-PDCAAS and the *p* value was found to 0.00128 (*p* < 0.05) wherein F > Fcrit (10.71 > 3.68), indicating that the samples were significantly different to each other with variable amino acid and k-PDCAAS score. Additionally, following the Tukey–Kramer procedure a comparison test was conducted and it was observed that the k-PDCAAS was significantly different than their in vitro digestibility and amino acid score for the six seaweed species wherein the absolute difference value was greater than their critical range (0.483 > 0.279 and 0.401 > 0.279).

### 3.2. Limiting Amino Acids, Essential Amino Acids, and Other Important Amino Acids Found These Six Seaweeds

The k-PDCAAS assessment method also helps to identify the first limiting amino acids for each seaweed tested. For the brown seaweeds, the identified limiting amino acids were L-tyrosine + L-phenylalanine for *A. esculenta*, L-histidine for *F. serratus* and L-leucine for *F. vesiculosus*. For *P. palmata* and *A. taxiformis* the identified limiting amino acids were L-tyrosine + L-phenylalanine and L-cysteine + L-methionine, respectively. For the green seaweed *U. latuca* L-cysteine and L-methionine were the observed limiting amino acids. The essential amino acids were found in all the seaweeds. However, as shown in Table 2 the concentration of the identified essential amino acids from the k-PDCAAS assessment were significantly lower where indicated and therefore are considered limiting amino acids for the individual seaweeds.

The most abundant amino acid found in *A. esculenta* was L-leucine (0.71 ± 0.004 g/100 g), followed by L-valine (0.54 ± 0.002 g/100 g), L-lysine (0.53 ± 0.002 g/100 g), and L-histidine (0.50 ± 0.01 g/100 g). For *F. serratus*, the most abundant essential amino acids were L-leucine (0.57 ± 0.01 g/100 g), followed by L-valine (0.56 ± 0.018 g/100 g), L-threonine (0.50 ± 0.005 g/100 g), and L-lysine (0.40 ± 0.02 g/100 g). Similarly, for *F. vesiculosus* abundantly occurring essential amino acids included L-histidine (0.57 ± 0.002 g/100 g), L-lysine (0.18 ± 0.08 g/100 g), and L-methionine (0.166 ± 0.05 g/100 g). In the case of *U. lactuca* L-valine (0.60 ± 0.004 g/100 g), L-leucine (0.58 ± 0.008 g/100 g), and L-threonine (0.46 ± 0.002 g/100 g) were the most abundant amino acids. For *P. palmata* L-valine (0.62 ± 0.008 g/100 g), L-leucine (0.59 ± 0.002 g/100 g), and L-threonine (0.47 ± 0.03 g/100 g) were present in the greatest quantities and for *A. taxiformis*, L-valine (0.95 ± 0.024 g/100 g), L-leucine (0.91 ± 0.02 g/100 g), and L-threonine (0.76 ± 0.034 g/100 g) were predominant. It is noteworthy that lysine lacking in plant based protein sources such as grains and legumes [36] was found in all the six seaweeds and was present in sufficient quantities in *A. esculenta* and *F. serratus*. The recommended daily allowance (RDA) for EAA per gram of protein intake includes 18 mg of histidine; 25 mg of isoleucine, methionine and cysteine, 55 mg of leucine, 51 mg of lysine, 47 mg of phenylalanine and tyrosine, 27 mg of threonine, 7 mg of tryptophan, and 32 mg of valine [12,38].

The amino acids L-glutamic acid, L-glycine, L-aspartic acid, L-alanine and L-arginine were also found in all the six tested seaweeds. This complies with previous research carried out by Terriente-Palacios and Castellari (2022), which reported that seaweeds are rich in the amino acids glutamic acid, aspartic acid, glycine and alanine thus providing umami flavor to foods [39].

Interestingly, taurine was found in four of the six seaweeds including in *A. esculenta* (0.434 ± 0.05 g/100 g), *F. vesiculosus* (3.97 ± 0.25 g/100 g), *P. palmata* (0.304 ± 0.007 g/100 g) and *A. taxiformis* (0.297 ± 0.03 g/100 g). Taurine also known as 2-aminoethanosulfonic acid is a conditionally essential amino acid that has an important function in brain, retina, heart, and blood cells [40,41]. Recently, taurine has been pointed out as a promising new therapeutic agent in the treatment of diseases affecting the muscles, central nervous system, cardiovascular system, and other vital metabolic disorders [41]. Terriente-Palacios and Castellari (2022), reported the taurine concentration in *P. palmata* at 0.55 ± 0.10 g/100 g dry weight and in our study we found a similar concentration 0.304 ± 0.007 g/100 g [40]. Overall, the amino acid composition of the seaweeds suggests that these seaweeds would be a suitable protein source for human nutrition providing both essential and non-essential amino acids.

## 4. Future Direction

When the three classes of seaweeds were compared for their in vitro protein digestibility, k-PDCAAS, and amino acid profile, the red and brown seaweeds, particularly *Palmaria palmate*, *Fucus serratus* and *Alaria esculenta*, were found to be the most suitable seaweeds, with the highest k-PDCAAS score. The protein content of all the analyzed seaweeds (ranging from 5.37 to 9.96%) were in agreement with the literature data [12,40] and was comparable to that of high-protein plant foods such as soybean. Astorga-España et al. reported that by consuming brown, red and green seaweeds, approximately 7.5–9.1% (brown), 10.9–13.3% (red), and 12–14.6% (green) of the daily recommended protein intake for men and women can be achieved [40,42]. Future work should include assessment of these seaweeds using the in vivo PDCAAS and DIAAS methods.

## 5. Conclusions

Of the seaweeds examined, *P. palmata*, *F. serratus* and *A. esculenta* had the highest k- PDCAAS values of 0.69 ± 0.014, 0.63 ± 0.084, 0.59 ± 0.021, respectively, and amino acid score values of 0.883 ± 0.019; 0.825 ± 0.108; 0.751 ± 0.022, respectively. Although the in vitro protein digestibility of each seaweed was similar, the k-PDCAAS and amino acid score values varied significantly for each seaweed. The seaweeds were found to be rich in essential amino acids and could satisfy the FAO RDA requirements for proteins. Additionally, the seaweeds were also found to contain other important amino acids such as taurine.

## Figures and Tables

**Table 1:** In vitro digestibility, k-PDCAAS, percentage protein and amino acid score values for six Irish seaweeds.

Sample Name	In Vitro Digestibility	Amino Acid Score	K-PDCAAS	Crude Protein (%)
Brown seaweeds				
*Alaria esculenta*	0.78 ± 0.002	0.751 ± 0.022	0.59 ± 0.021	9.96 ± 0.51
*Fucus serratus*	0.77 ± 0.001	0.825 ± 0.108	0.63 ± 0.084	6.122 ± 1.18
*Fucus vesiculosus*	0.82 ± 0.001	0.101 ± 0.002	0.08 ± 0.013	9.02 ± 0.26
Green seaweed				
*Ulva lactuca*	0.79 ± 0.003	0.184 ± 0.012	0.15 ± 0.014	5.37 ± 0.74
Red seaweeds				
*Palmaria palmata*	0.78 ± 0.002	0.883 ± 0.019	0.69 ± 0.014	7.78 ± 0.42
*Asparagopsis taxiformis*	0.79 ± 0.001	0.393 ± 0.01	0.31 ± 0.01	7.523 ± 0.02

All average values are represented with standard deviation based on two individual analyses. Significant difference between samples were observed for amino acid score and K-PDCAAS, *p* < 0.05; *p* value: 0.00435, 0.00128.

**Table 2:** Amino acid profiles of the six seaweeds and ratios of the amino acid corresponding to the reference protein.

Amino Acids	*Alaria Esculenta*		*Fucus Serratus*		*Fucus Vesiculosus*		*Ulva Lactuca*		*Palmaria Palmata*		*Asparagopsis Taxiformis*	
	Total AA (g/100 g CP)	Ratio	Total AA (g/100 g CP)	Ratio	Total AA (g/100 g CP)	Ratio	Total AA (g/100 g CP)	Ratio	Total AA (g/100 g CP)	Ratio	Total AA (g/100 g CP)	Ratio
L-Cys + L-Met	0.35 ± 0.001	1.18 ± 0.001	0.15 ± 0.0001	0.92 ± 0.0001	0.89 ± 0.009	3.30 ± 0.009	0.02 ± 0.001	0.11 ± 0.001	0.49 ± 0.002	1.55 ± 0.002	0.07 ± 0.0002	0.25 ± 0.0002
L-Trp	0.0 ± 0.0	0.0 ± 0.0	0.0 ± 0.0	0.0 ± 0.0	0.0 ± 0.0	0.0 ± 0.0	0.0 ± 0.0	0.0 ± 0.0	0.0 ± 0.0	0.0 ± 0.0	0.0	0.0 ± 0.0
L-Hydroxy Pro	0.0 ± 0.0	-	0.0 ± 0.0	-	0.0 ± 0.0	-	0.08 ± 0.001	-	0.0 ± 0.0	-	0.0 ± 0.0	-
L-Asp	0.99 ± 0.002	-	0.98 ± 0.03	-	0.47 ± 0.02	-	0.94 ± 0.05	-	1.03 ± 0.006	-	1.50 ± 0.10	-
L-Thr	0.46 ± 0.009	1.15 ± 0.009	0.50 ± 0.005	2.19 ± 0.03	0.15 ± 0.013	0.41 ± 0.01	0.46 ± 0.002	1.63 ± 0.001	0.47 ± 0.03	1.10 ± 0.03	0.76 ± 0.034	1.88 ± 0.034
L-Ser	0.51 ± 0.02	-	0.63 ± 0.012	-	0.07 ± 0.0007	-	0.66 ± 0.001	-	0.51 ± 0.001	-	1.02 ± 0.048	-
L-Glu	1.41 ± 0.001	-	0.57 ± 0.01	-	1.71 ± 0.01	-	0.78 ± 0.003	-	1.52 ± 0.013	-	1.27 ± 0.05	-
L-Pro	0.48 ± 0.006	-	0.36 ± 0.017	-	0.34 ± 0.001	-	0.44 ± 0.01	-	0.44 ± 0.004	-	0.62 ± 0.008	-
L-Gly	0.53 ± 0.001	-	1.40 ± 0.08	-	0.17 ± 0.005	-	1.39 ± 0.05	-	0.58 ± 0.031	-	2.09 ± 0.02	-
L-Ala	0.63 ± 0.009	-	1.42 ± 0.02	-	0.46 ± 0.03	-	1.50 ± 0.04	-	0.63 ± 0.003	-	2.01 ± 0.06	-
L-Val	0.54 ± 0.002	1.31 ± 0.002	0.56 ± 0.018	2.43 ± 0.017	0.12 ± 0.001	0.32 ± 0.001	0.60 ± 0.004	2.02 ± 0.003	0.62 ± 0.008	1.40 ± 0.008	0.95 ± 0.024	2.29 ± 0.02
L-Ile	0.43 ± 0.03	1.30 ± 0.03	0.34 ± 0.01	1.85 ± 0.01	0.12 ± 0.001	0.39 ± 0.001	0.33 ± 0.006	1.39 ± 0.006	0.36 ± 0.001	1.04 ± 0.001	0.62 ± 0.01	1.87 ± 0.01
L-Leu	0.71 ± 0.004	0.91 ± 0.004	0.57 ± 0.01	1.30 ± 0.01	0.06 ± 0.001	0.08 ± 0.001	0.58 ± 0.008	1.03 ± 0.008	0.59 ± 0.002	0.71 ± 0.002	0.91 ± 0.02	1.16 ± 0.02
L-Tyr + L-Phe	0.47 ± 0.001	0.63 ± 0.001	0.33 ± 0.01	0.78 ± 0.01	0.85 ± 0.01	1.24 ± 0.01	0.31 ± 0.009	0.58 ± 0.009	0.43 ± 0.006	0.55 ± 0.006	0.46 ± 0.006	0.62 ± 0.006
L-Lys	0.53 ± 0.002	0.77 ± 0.002	0.40 ± 0.02	1.05 ± 0.02	0.18 ± 0.08	0.27 ± 0.08	0.29 ± 0.009	0.58 ± 0.009	0.56 ± 0.01	0.77 ± 0.01	0.46 ± 0.01	0.67 ± 0.01
L-His	0.50 ± 0.01	2.25 ± 0.01	0.10 ± 0.0002	0.76 ± 0.0002	0.57 ± 0.002	2.77 ± 0.002	0.08 ± 0.002	0.47 ± 0.002	0.47 ± 0.015	1.95 ± 0.015	0.11 ± 0.01	0.48 ± 0.01
L-Arg	0.70 ± 0.015	-	0.25 ± 0.009	-	0.12 ± 0.031	-	0.27 ± 0.002	-	0.60 ± 0.001	-	0.38 ± 0.014	-

All average values are represented with standard deviation based on two individual analyses. Significant difference between samples were observed for amino acid profile, *p* < 0.05; *p* value: 0.020.

**Table 3:** Comparison of k-PDCAAS values referenced for plants compared to tested seaweeds.

Food Materials	PDCAAS	Ref
Vegetables	0.73	[32]
Fresh and dry fruits	0.64	[32]
Soy	0.91	[31]
Wheat	0.42	[31]
Rice	0.81	[31]
Chickpeas	0.62–0.65	[33]
Barley	0.44–0.53	[33]
*Porphyra columbina* (red seaweed)	0.327	[29]
*Palmaria palmata* (red seaweed)	0.69	In this study
*Asparagopsis taxiformis* (red seaweed)	0.31	In this study
*Alaria esculenta* (brown seaweed)	0.59	In this study
*Fucus serratus* (brown seaweed)	0.63	In this study
*Fucus vesiculosus* (brown seaweed)	0.08	In this study
*Ulva lactuca* (green seaweed)	0.15	In this study

## Data Availability

The datasets generated for this study are available on request to the corresponding author.
